# Changes in major psychiatric disorders in children and adolescents from 2001 to 2020: A retrospective single-center study

**DOI:** 10.3389/fpsyt.2022.1079456

**Published:** 2023-01-09

**Authors:** Hongyu Zheng, Xiaolu Jiang, Rong Yang, Shuo Wang, Hui Zhong

**Affiliations:** ^1^Department of Child and Adolescents, Affiliated Psychological Hospital of Anhui Medical University, Hefei, Anhui, China; ^2^Anhui Mental Health Center, Hefei, Anhui, China; ^3^School of Mental Health and Psychological Sciences, Anhui Medical University, Hefei, Anhui, China; ^4^Department of Child and Adolescents, Hefei Fourth People’s Hospital, Hefei, Anhui, China

**Keywords:** hospitalization trends, psychosis, adolescent patients, age trends, gender trends

## Abstract

**Objective:**

This study aimed to determine the hospitalization rates, length of stay, age at the time of admission, and sex distribution for major psychiatric disorders in children and adolescents and provide a reference for early intervention for these diseases and distribution of medical resources in hospitals.

**Methods:**

We screened 4,423 patients in the child and adolescent wards of the Anhui Provincial Mental Health Center from 2001 to 2020, and examined the top four (81.1%) mental health disorders that accounted for the overall proportion of patients admitted, namely schizophrenia (SCZ) (45.7%), depressive disorder (DD) (14.5%), bipolar disorder (BD) (9.3%), and childhood emotional disorder (CED) (11.6%), and for each disorder, the percentage of hospitalization, length of stay, age at admission, and sex distribution were analyzed.

**Results:**

From 2001 to 2020, there was a significantly decreasing trend in the proportion of hospitalizations for SCZ (*p* < 0.001) and an increasing trend for depression and CED (*p* < 0.001). In terms of length of stay, SCZ was significantly longer than the other three disorders (*p* < 0.001), whereas there was no significant difference between DD, BD, and CED, and there was no significant trend in length of stay for any of the four disorders. The age at admission for CED was significantly lower than that for the other three disorders (*p* < 0.001). There was a decreasing trend in the age at admission for DD (*p* = 0.011) and an increasing trend for BD (*p* = 0.001). A significant increase in the number of female patients admitted for SCZ, DD, and CED was observed, while there was no significant change in the sex ratio for BD.

**Conclusion:**

Although there is a significant downward trend in the percentage of hospitalizations for SCZ, it is still the most common psychiatric disorder in children and adolescents. We observed a significant increase in the percentage of hospitalizations for DD and CED. In addition, the proportion of female patients being hospitalized is on the rise, and this aspect requires continuous attention.

## 1. Introduction

A recent study reported an overall prevalence of 17.5% of mental disorders among children aged 6–16 years in China (1). Similarly, in an earlier multi-country study, it was found that at least 11–16% of children and adolescents experience one or more mental disorders (2). Early diagnosis and treatment have a positive impact on the prognosis and are part of the primary and secondary prevention approaches, but unfortunately, many of these children and adolescents do not receive effective diagnosis and treatment (3–5). This poses a great challenge in the quest for the treatment of mental disorders in children and adolescents.

A study in the UK noted that 1 in 4 people experience a mental health and/or substance use disorder at some point in their lives (6), and the prevalence of common mental disorders had increased by 2% in children from 1999 to 2017 and 2% in adults from 1993 to 2014 (7, 8). However, only 0.2% of the adults with poor mental health and 1.8% with severe mental health disorders receive inpatient care (7).

Studies have estimated that China accounts for approximately 17% of the global burden of mental disorders (9), and with the development of society, mental disorders have likewise attracted the attention of the Chinese population and the government, with child and adolescent mental illnesses receiving increasing attention from society; for example, on December 19, 2019, China’s National Health and Wellness Commission, in conjunction with multiple national departments, formulated the Health China Action–Action Plan for Children and Youth Mental Health, which specifically states that a social environment conducive to children and youth mental health should be basically built, forming schools, communities, families, media, medical and health institutions The program specifically states that a social environment conducive to the mental health of children and adolescents should be basically built, and a mental health service model should be formed with the linkage of schools, communities, families, media and medical and health institutions. On September 11, 2020, the National Health and Wellness Commission of China proposed the inclusion of depression screening in student health examinations at high schools and universities. Meanwhile, on September 8, 2021 and October 29, 2021, the National Health and Wellness Commission issued the “China’s Child Development Program” and the “Healthy Child Action Enhancement Plan” respectively, both of which proposed to strengthen mental health services for children.

The prevalence of mental disorders is often not correlated with hospitalization rates, and a British study reported that for anxiety disorders, hospitalization rates almost doubled from 1998 to 2020, while the prevalence of anxiety disorders identified by population surveys showed an increase of only 2% from 1997 to 2017 (2). Similarly, in a study by Brazilian scholars published in 1997 on the prevalence of mental disorders in a community sample (10), the most common diagnosis was phobia (14.1%), followed by depressive states (10.2%), anxiety disorders (9.6%), and alcoholism/dependence (9.2%). Another study on hospitalization rates in Brazil in 2020 showed depressive states, alcohol-related, and psychiatric disorders as the main causes of hospitalization (11). Therefore, although in an epidemiological survey in our country attention deficit hyperactivity disorder (ADHD), oppositional defiant disorder (ODD), and major depressive disorder (MDD) were listed as the most common psychiatric disorders in children and adolescents (1), it is reasonable to doubt that this result carries over to the hospitalization rates of child and adolescent for psychiatric disorders.

From a clinical perspective, hospitalization rates for mental disorders may be largely reflective of the severity of mental disorders and their impact on society and the wellbeing of citizens. Epidemiological surveys of mental disorders in children and adolescents have been systematically studied in many countries, providing useful information for mental health service providers and government officials; however, definitive studies on hospitalization due to mental disorders in adolescent children are limited; hence, coinciding with the repeated national emphasis on strengthening mental health services for children and adolescents, this study hopes to provide additional information to medical professionals and government personnel from the perspective of hospitalization, as well as to provide a reference for the allocation of medical resources in psychiatric hospitals through a study of patients hospitalized in the child and adolescent wards of our center over the past 20 years.

## 2. Materials and methods

Located in Hefei, Anhui Province, China, our center serves patients with mental illness in 16 prefecture-level cities in Anhui Province. Anhui Province is located in the Yangtze River Delta region of East China, between 114°54′–119°37′ E longitude and 29°41′–34°38′ N latitude. The terrain consists of plains, hills and mountains, with a resident population of about 61.13 million in 2021 and an urbanization rate of 59.4% of the population, it is a It is a populous province with mainly agriculture and underdeveloped industry and commerce, and its economic level in 2021 is in the middle level of 34 provinces and cities in China.

This study was approved by our local ethics committee, and data were obtained through the case management system of Anhui Mental Health Center with the following inclusion criteria:

(1) Patients admitted to child and adolescent wards.
(2) Patients admitted from January 1, 2001 to December 31, 2020.
(3) Patients aged <18 years at the time of admission.

Because this study included all inpatients admitted to the pediatric adolescent ward of our center over a 20-year period, with a large time span and previous disease diagnostic criteria including ICD-9, CCMD-3, DSM-4, DSM-5, and ICD-10, this study standardized the discharge diagnosis according to the current ICD-10 diagnostic criteria:

• F00–F09: Organic, including symptomatic, mental disorders
• F10–F19: Mental and behavioral disorders due to psychoactive substance use
• F20–F29: Schizophrenia, schizotypal and delusional disorders
• F30–F39: Mood (affective) disorders
• F40–F48: Neurotic, stress-relataed and somatoform disorders
• F50–F59: Behavioral syndromes associated with physiological disturbances and physical factors
• F60–F69: Disorders of adult personality and behavior
• F70–F79: Mental retardation
• F80–F89: Disorders of psychological development
• F90–F98: Behavioral and emotional disorders with onset usually occurring in childhood and adolescence
• Other conditions (e.g., Unclear diagnosis) (OT).

To prevent the inclusion of so many diseases that the analysis would be too cumbersome and unrepresentative, we only addressed the most prevalent diseases in the subsequent analysis.

Information on age at admission, sex, and length of stay were also collected. Those with an unclear diagnosis at discharge were not included in the follow-up analysis.

### 2.1. Statistical analysis

Inpatient share calculation method:

Percentage of total hospitalizations for the disease over 20 years = total number of patients hospitalized for the disease over 20 years/total number of patients hospitalized in the ward over 20 years × 100%

Percentage of hospitalization per year = total number of patients hospitalized for a particular disease in that year/total number of patients hospitalized in the ward in that year × 100%

Female gender share calculation method:

Female gender ratio per year = total number of female patients hospitalized for a disease in that year/total number of patients hospitalized for that disease in the ward in that year × 100%

Statistical analysis was performed using SPSS 25.0, with $\,\bar{\text{x}}$± s description for quantitative data, ANOVA for group comparisons, Shapiro–Wilk test, and Skewness and Kurtosis analysis to check for normal distribution. In case of violation of the normality assumption, the Jonckheere–Terpstra test was used for temporal analysis to maintain the sequential character of the variables “year”; if a correlation was observed, Kendall’s τ-b correlation coefficient was calculated.

## 3. Results

A total of 4,423 patients aged less than 18 years were admitted to the pediatric adolescent ward of our center from 2001 to 2020, and the top four (81.1%) disorders that accounted for the overall proportion of admissions were analyzed in this study, namely schizophrenia (SCZ) (45.7%), depressive disorder (DD) (14.5%), bipolar disorder (BD) (9.3%), and childhood emotional disorder (CED) (11.6%). Among them, 2,234 patients (50.5%) were male. Disorders not included in this study were organic mental disorder (1.2%), obsessive-compulsive disorder (3.2%), dissociative (conversion) disorder (3%), hyperactivity disorder (0.2%), and other mental disorders. A summary of demographic characteristics regarding sex distribution, mean age at admission, and mean length of hospitalization (LOS) of the analyzed population is presented in Table 1.

**TABLE 1:** Demographic characteristics of the population.

	Female (% *n*)	Male (% *n*)	Mean age, years (SD)	Mean ELOS, days (SD)
SCZ (F20)	987 (48.8%)	1,035 (51.2%)	15.61 (2.13)	56.75 (50.39)
DD (F32–F33)	422 (65.9%)	218 (34.1%)	15.59 (1.67)	37.06 (24.09)
BD (F31)	190 (46.2%)	221 (53.8%)	15.30 (2.33)	36.60 (28.35)
CED (F93.9)	235 (45.9%)	277 (54.1%)	13.63 (2.14)	35.24 (24.67)
F00–F09	19 (35.8%)	34 (64.2%)	13.89 (2.80)	30.38 (29.59)
F10–F19	0 (0%)	1 (100%)	16	138
F21–F29	44 (50.0%)	44 (50.0%)	14.98 (2.28)	31.15 (24.85)
F30, F34–F39	20 (35.7%)	36 (64.3%)	15.93 (1.54)	39.16 (18.68)
F40–F41	28 (50%)	28 (50%)	15.73 (3.02)	35.64 (23.94)
F42	39 (27.5%)	103 (72.5%)	15.77 (2.13)	49.18 (27.24)
F43	25 (55.6%)	20 (44.4%)	14.07 (2.67)	27.16 (21.84)
F44	88 (65.7%)	46 (34.3%)	13.84 (3.02)	28.28 (21.71)
F45–F48	16 (35.6%)	29 (64.4%)	16.71 (1.58)	42.16 (20.83)
F50–F59	20 (55.6%)	16 (44.4%)	14.89 (2.42)	29.50 (22.95)
F60–F69	2 (18.2%)	9 (81.8%)	15.18 (1.89)	21.18 (14.59)
F70–F79	42 (50.7%)	41 (49.3%)	12.71 (3.05)	40.65 (26.74)
F80–F89	0 (0%)	10 (100%)	9.70 (4.00)	28.00 (20.74)
F90–F93.8, F94–F98	9 (13.6%)	57 (86.4%)	12.56 (3.27)	36.95 (26.98)
OT	3 (25.0%)	9 (75.0%)	13.92 (3.58)	21.17 (23.71)
Total	2,189 (49.5%)	2234 (50.5%)	15.14 (2.37)	45.23 (33.67)

### 3.1. Percentage of hospitalization

**FIGURE 1:**
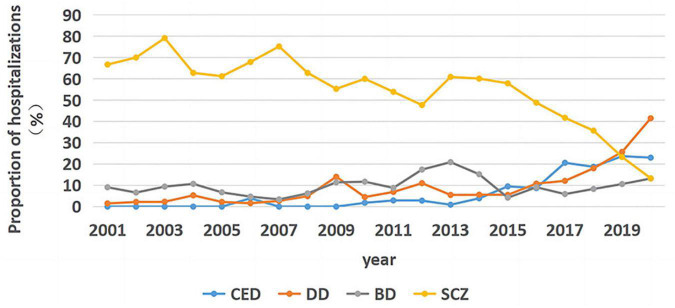
Trends in hospitalization rates for the four major diseases from January 2001 to December 2020.

Jonckheere–Terpstra test was used to test the trends for each disease (Figure 1). A statistically significant trend was observed in the percentage of hospitalizations for SCZ (Tj-t = 25.5, *z* = −4.512, *p* < 0.001), and the correlation coefficient for SCZ was estimated using Kendall’s τ-b correlation coefficient (rτ = −0.734, *p* < 0.001). A statistically significant change was observed in the percentage of hospitalizations for DD (Tj-t = 163.0, *z* = 4.417, *p* < 0.001), and the correlation coefficient for DD was estimated using Kendall’s τ-b correlation coefficient (rτ = 0.720, *p* < 0.001). A statistically non-significant trend was observed for BD (Tj-t = 110.5, *z* = 1.006, *p* = 0.314), while a statistically significant trend was observed for CED (Tj-t = 161.5, *z* = 4.474, *p* < 0.001), and the correlation coefficient for CED was estimated using Kendall’s τ-b correlation coefficient (rτ = 0.760, *p* < 0.001).

### 3.2. Length of hospitalization

**FIGURE 2:**
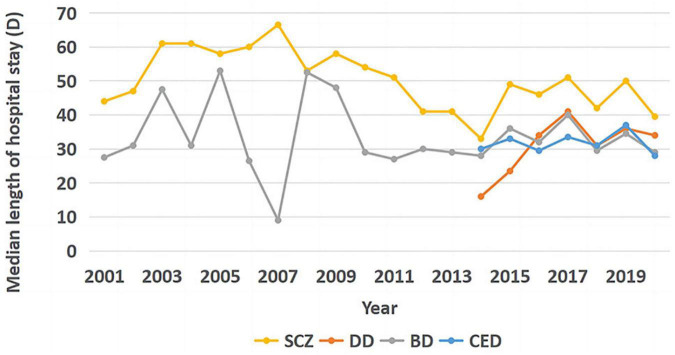
Trends in median length of stay for the four diseases.

Kruskal–Wallis *H*-test was performed to determine whether the LOS values were evenly distributed between each cluster. Differences among median LOS values for the conditions were statistically significant (*X*^2^ = 294.718, *p* < 0.001). Pairwise comparisons were performed using Bonferroni correction for multiple comparisons. The statistical significance was set at *p* < 0.001; the LOS was significantly longer for SCZ than for the other three disorders (*p* < 0.001), whereas no significant difference was observed in the LOS among the other three disorders (DD-CED: *p* = 0.142, DD-BD: *p* = 0.318, BD-CED: *p* = 1.000). As seen in Figure 2, no significant trend was observed in the LOS for SCZ (Tj-t = 56.0, *z* = −2.536, *p* = 0.011).

Considering that there were very few inpatient admissions for DD before 2014, only the inpatient days for DD from 2014 to 2020 were analyzed. No significant trend was observed in inpatient days for DD (Tj-t = 15.5, *z* = 1.519, *p* = 0.129). No significant trend was observed in hospitalization days for BD (Tj-t = 96.0, *z* = 0.065, *p* = 0.948). Considering that too few inpatients were diagnosed with CED before 2014, only the inpatient days for CED from 2014 to 2020 were analyzed and no significant trend was observed in hospitalization days for CED (Tj-t = 11.0, *z* = 0.150, *p* = 0.881).

### 3.3. Age at admission

The Kruskal–Wallis *H*-test was used for pairwise comparisons with Bonferroni correction for multiple comparisons. The statistical significance was set at *p* < 0.001; the age at admission was significantly lower for CED than for the other three disorders (*p* < 0.001), whereas there was no significant difference in age at admission among the other three disorders (SCZ-DD: *p* = 0.322, SCZ-BD: *p* = 0.108, DD-BD: *p* = 1.000).

**FIGURE 3:**
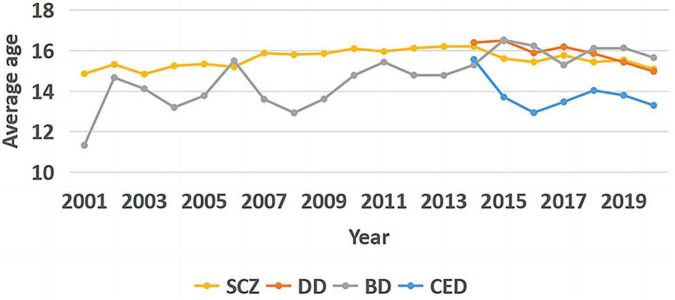
Trend graph of mean age at hospitalization for the four diseases.

As seen in Figure 3, no significant trend was observed in age at admission for SCZ (Tj-t = 118.0, *z* = 1.494, *p* = 0.135). A significant downward trend was noted in age at admission for DD from 2014 to 2020 (Tj-t = 2.0, *z* = −2.553, *p* = 0.011), and the correlation coefficient for DD was estimated using Kendall’s τ-b correlation coefficient (rτ = −0.810, *p* = 0.011).

A significant upward trend was observed in age at admission for BD (Tj-t = 148.0, *z* = 3.443, *p* = 0.001), and the correlation coefficient for BD was estimated using Kendall’s τ-b correlation coefficient (rτ = 0.561, *p* < 0.001). No significant trend was noted in age at admission for CED from 2014 to 2020 (Tj-t = 8.0, *z* = 0.188, *p* = 0.851).

### 3.4. Inpatient sex distribution

**FIGURE 4:**
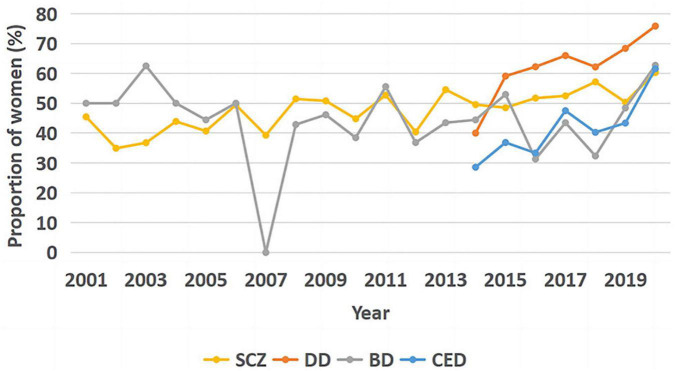
Trend graph of the percentage of female patients hospitalized for the four diseases.

As shown in Figure 4, there was a significant upward trend in the gender share of schizophrenia hospitalizations for females (Tj-t = 147.0, *z* = 3.374, *p* = 0.001); the correlation coefficient was estimated using Kendall’s τ-b correlation coefficient (rτ = 0.547, *p* = 0.001).

A significant upward trend was observed in the proportion of female inpatients with DD (Tj-t = 19.0, *z* = 2.553, *p* = 0.011); the correlation coefficient was estimated with Kendall’s τ-b correlation coefficient (rτ = 0.810, *p* = 0.011). No significant trend was observed in the proportion of female inpatients with BD (Tj-t = 81.0, *z* = −0.914, *p* = 0.361). An upward trend was noted in the proportion of female inpatients with CED (Tj-t = 18.0, *z* = 2.253, *p* = 0.024); the correlation coefficient was estimated with Kendall’s τ-b correlation coefficient (rτ = 0.714, *p* = 0.024).

## 4. Discussion

In the current study, we selected the top four disorders accounting for the highest percentage of hospitalizations of 4,423 inpatients in the pediatric and adolescent wards at our center for analysis. To the best of our knowledge, this may be the first study to provide an epidemiological account of adolescent inpatients with the primary inpatient diagnosis of psychiatric disorders in China over a 20-year period. In this study, we noted several findings: (1) a decrease in the hospitalization rate of patients with non-emotional psychiatric disorders and an increase in the hospitalization rate of patients with emotional psychiatric disorders; (2) no significant trend in the duration of hospitalization of patients with psychiatric disorders over 20 years; and (3) a significant increase in the proportion of female patients hospitalized for both affective and non-affective psychiatric disorders, with the exception of BD.

In a previous survey on the prevalence of psychiatric disorders in children and adolescents in China, it was noted that ADHD, ODD, and MDD were the most common psychiatric disorders in children and adolescents (1), and the guidelines for the treatment of disorders indicate that patients with ADHD and ODD require more comprehensive treatment with medication in conjunction with family and societal support than hospitalization. Therefore, the number of patients hospitalized for these disorders in our sample was very low, and when we excluded these, MDD was the most common mental disorder, which is consistent with our results. This result is consistent with the results of a study from Brazil (11), where depression and alcohol-related psychiatric disorders were the main causes of hospitalization.

Our data showed a decrease in the percentage of hospitalizations for SCZ, and considering that the number of beds in the pediatric/adolescent ward of our center was expanded from 40 to 80 beds in 2014 and that we used the hospitalization rate rather than the number of hospitalizations, we consider the decrease in the hospitalization rate for SCZ to be meaningful, a result that is also consistent with those of previous studies. Ostinelli et al. (12) reported a decreasing trend in hospitalization rates for SCZ over 15 years in Milan, Italy, although their study focused on young patients (<35 years). Likewise, another finding of that report is consistent with our results, which is an increase in hospitalization rates for affective psychoses. This trend was reported in many studies that were not age-restricted (13–15). However, in a UK study (16), it was noted that only the prevalence rates of anxiety and eating disorders increased and those of all other psychiatric disorders decreased in the unrestricted age context. However, for children and adolescents, hospitalization for DD continued to increase by 5.6% between 1998 and 2019. However, this is inconsistent with data from the National Bureau of Statistics, where the percentage of hospitalizations for patients younger than 14 years old was around 1.2% in the last 5 years among patients hospitalized for schizophrenia and did not change significantly.

The decline in hospitalization rates for SCZ may be related to the enhancement of outpatient and community treatment, as our center has added special outpatient clinics specifically for pediatric and adolescent patients, while the state has been actively improving community treatment for major mental illnesses in recent years, and in China, patients with SCZ, as a chronic illness, are able to receive state financial subsidies, along with improved national health insurance policies. The decline in hospitalization rates for SCZ cannot be attributed to the lack of financial resources among the patients’ families. Certainly, the partial success of the anti-stigma campaign for the illness, especially in the case of affective disorders, partly explains our reported results, although a meta-analysis of studies in mainland China reported that the local scores for the level of stigma for mental disorders ranged from 60 to 80% (17), which are rather high.

The high hospitalization rates for SCZ in the earlier years can be attribute to the low awareness among the population about affective psychiatric disorders, such as depression or BD, and people may not consider affective psychiatric disorders as diseases but rather mood changes. In addition, SCZ is a chronic and complex psychiatric disorder, and the patients often have a reduced ability to learn, work, take care of themselves, and maintain relationships and poor general life skills (18). The impact of SCZ on the family and society is more pronounced and can exert a significant social and economic burden on the patient’s family (19). SCZ is often accompanied by significant psychotic positive symptoms, such as hallucinations and delusions, which clearly distinguish it from other mental health disorders.

In our study, we observed a significant increase in the rate of hospitalization for affective psychiatric disorders after 2015, this is consistent with data from the National Bureau of Statistics, which shows that in the last 5 years, the number of patients younger than 14 years old with affective mental disorders increased from 1.7% in 2016 to 7.9% in 2020. One is the increase in awareness of affective mental disorders due to the national popularization of mental disorders, and the other may be due to the increasing attention to psychological problems of children and adolescents in the country, which requires schools to have psychological counseling teachers, as well as increasing the popularization of affective mental disorders in the society and strengthening the cooperation between psychiatric hospitals and schools and communities for early detection and early intervention.

Length of hospitalization did not show a significant change with time, which is inconsistent with previous reports (12, 16, 20); previous studies pointed out that the hospitalization duration was shortened because of the use of long-acting formulations of atypical antipsychotics, which are also currently widely used in our center; however, long-acting formulations are not widely prescribed to pediatric patients. The application of long-acting injections for the treatment of SCZ was recommended in the 2015 Chinese expert consensus report (21); however, a study conducted in 2020 on the application pattern of long-acting injections in SCZ in Asia reported that the application rate of long-acting injections in China is still low, at about 0.66% (22). Currently, no indication for the use of antipsychotic long-acting injections for treating SCZ in children or adolescents are described (23), despite a growing body of literature supporting their use from the early stages of the disease for reducing relapse (24–27). This could be the reason for the discrepancy between our results and those of previous studies. However, the LOS for SCZ in the present study (mean LOS, 56.75 days), is similar to that reported in some studies, which is 30–45 days (28). The mean LOS for SCZ in Germany was 50.2 days (29). A Chinese study also indicated that 20–50 days may be the optimal LOS for SCZ (30). This variation may be closely related to local mental health policies and health insurance, and according to previous studies, the LOS in psychiatric inpatient wards varies widely, with the average LOS ranging from 20 to 740 days (31, 32). Also in reviewing the Chinese Health and Health Statistical Yearbook for the past 5 years, it was found that the average psychiatric hospitalization days in China increased from 51.7 days in 2016 to 56.5 days in 2020, and the average hospitalization days on the schizophrenia spectrum increased from 52.04 days in 2016 to 56.31 days in 2020, which is generally consistent with the data of our center, but our center did not show a significant change. Also in we found a significant difference, compared with the national statistics of China in the last 5 years, the average length of stay in our center for affective mental disorders did not change significantly, the average length of stay for DD, BD, and CED were around 35 days, while the national statistics showed that the average length of stay for affective mental disorders decreased from 20.68 days in 2016 to 18.85 days in 2020 One possible reason for this is that the national statistics are provided for national and all-age patients, and due to health insurance policies and hospital policies, some hospitals have certain requirements for ward rotation rates (i.e., bed utilization rates), requiring wards to speed up bed turnover to be able to provide medical services to more patients and provide financial support to hospitals; another reason may be that families pay more attention to children and adolescents with affective disorders than adult patients with affective disorders, and prefer to discharge their children in a better condition rather than being discharged for outpatient treatment when their condition is temporarily stabilized.

Regarding age at admission, this study reported a significantly lower age at admission for CED than for the other three disorders, and we also studied a few relevant reports on CED, which may be related to the sociocultural environment, where industrialization and urbanization over the past 40 years have dramatically changed the Chinese society and negatively affected the mental health of many children and adolescents (33). Patients with CED in China show irritability, difficulty adapting to certain environmental changes, and mood changes in early adolescence that are difficult to explain by disorders such as depression, anxiety, or adjustment disorders. In China, CED is more likely to exist as a transitional diagnosis for those patients with early onset, especially the first onset, whose symptoms often fail to meet the diagnostic criteria of SCZ or other affective psychiatric disorders due to atypicality or complexity of symptoms. Moreover, CED is more acceptable to Chinese parents than stigmatizing diagnoses such as SCZ or depression. From our data, there is no significant change in the age of admission for schizophrenia, but there is a decreasing trend in the age of admission for DDs, which is similar to the data from the National Bureau of Statistics, which shows that from 2016 to 2020, there is no significant change in the percentage of patients younger than 14 years old in schizophrenia, regardless of male or female, but in affective mental disorders, the percentage of male less than 14 years old patients increased from 2.1% in 2016 to 4.7% in 2020, while the rising trend was more pronounced for female patients, with the percentage of female patients younger than 14 years old increasing from 1.4 in 2016 to 9.6% in 2020, which indirectly reflects the increase in the proportion of hospitalizations and the decrease in the age of hospitalization for affective psychoses. Although the magnitude of this decrease is very low, for example, DDs decreased from 16 to 15 years in 5 years, which may be related to the age limit of patients included in the study, it may be more meaningful to include all-age data, meanwhile, in recent years, the National Health Care Commission has proposed the need to improve the recognition rate of childhood affective disorders by psychiatrists, and also requested to strengthen the integration of medical-educational and improve the attention and recognition rate of childhood and adolescent affective disorders in schools, and secondly, it cannot be excluded that the decrease in age is related to the incidence tendency of low age of childhood and adolescent affective disorders in China in recent years, furthermore, parents are now more demanding of their children whilst being sensitive to changes in their children’s moods, resulting in timely awareness of abnormalities in children’s moods.

The increasing age at hospitalization for BD may be related to the characteristics of the disease. As per the ICD-10, for the diagnosis of BD, the patient must have depressive episodes as well as one or two manic episodes. Some patients choose our regional medical center when they are re-admitted, and the diagnosis of DD may have been made at the initial admission because of the presentation of depressive episodes or the diagnosis of CED may be revised to BD only after the presentation of one manic episode at the time of readmission. Previous studies have pointed out that BD with depressive phase as the first episode accounts for most patients diagnosed with BD (34–36), while in turn 25–60% or even more of patients with SCZ present with depressive symptoms (37–41). In addition, the depressive and manic phases of BD do not always alternate, so the delayed diagnosis can be delayed by 5–10 years or even longer (42–47). Another analysis (48) pointed out that the prospective diagnostic stability of SCZ spectrum and affective psychotic disorders is high, while in patients with other first-onset psychotic disorders, the diagnostic stability is low and most patients are classified as schizophrenic at the time of follow-up. This implies that a proportion of patients may have their diagnosis modified in subsequent treatment.

In our study, the proportion of female patients showed an elevated trend for SCZ, depression, and CED. SCZ onset at <18 years is referred to as early onset SCZ, and more males had early onset SCZ, which is a reflection of the characteristics of the disease itself, and some studies have shown that more male patients than female patients have childhood onset, with sex differences gradually equalizing with age (49). Another reason is that the symptoms of early SCZ are more atypical in females than in males, making them difficult to detect in time. An important reason may be the phenomenon of male preference over female, which directly and indirectly affects the investment in women’s health (50), and men are more valued as the main workforce when the level of economic development is poor. SCZ can cause deficits in memory and executive abilities, and these deficits can significantly affect patients’ daily behavior and ability to work, which not only makes them different from the norm, but can also have a significant impact on the patient’s family, so the public prefers aggressive treatment to undo the loss of the family workforce. Also a 2017 mate (51) analysis noted that earlier age of onset, more hospitalizations or length of stay, more negative symptoms, and worse prognosis may also contribute to the predominance of early male schizophrenia patients. Also the younger the age of onset, the worse the premorbid functioning of schizophrenia, the more severe the baseline illness, and the greater the impairment of social and occupational functioning later in life (52). However, a study in the USA noted a decrease in the hospitalization rates among women and higher medication compliance in women, while men presented with more negative symptoms and more severe psychiatric symptoms requiring hospitalization and high doses of antipsychotic medication (20). The proportion of depressed female patients also showed an increasing trend, which is in line with the perception that MDD is twice as common in women as in men, as reported in one study (53), a difference that has been attributed to psychological and physiological differences (54).

### 4.1. Limitations

The main limitation of our study is that limited data were available; therefore, other variables, such as medical comorbidities, family economic and cultural level, the severity of the symptoms, disease duration, and treatment options, could not be analyzed. The lack of these data prevented us from further studying the factors influencing the hospitalization.

Because of the large time span of the study, the criteria for diagnosis partially changed. The diagnosis of psychiatric disorders is based on symptomatic presentations, and the majority of these diagnoses are unlikely to be supported by the same diagnostic tools with high confidence validity, which makes the diagnoses highly heterogeneous. Therefore, standardization of previous diagnoses using ICD-10 diagnostic criteria may misclassify some patients to other groups, leading to misclassification bias. Our center is located in the interior of China, and the diagnostic criteria used are ICD-10, which may be different from those of other countries, and therefore may result in the same disease being diagnosed differently in two countries, which may be one of the reasons for the difference in data from some countries.

The trend graphs in this paper also show that some of the data have repeated fluctuations over 20 years, but the paper only makes overall trend judgments without more detailed analysis, for example, without further analysis of certain time points with significant changes. The prevalence and onset of disease may change from period to period, and these changes may have an impact on hospitalization for disease, which was not analyzed in more detail in this study. Also for this study we were unable to analyze data on whether the inpatient admissions were first-time, multiple admissions, and duration of untreated disease, so we lost the possibility of comparing with some of the studies.

Another shortcoming of this study is that there are few studies on the hospitalization of children and adolescents with psychiatric disorders in China, and we also did not search for studies on psychiatric hospitalization of children and adolescents in other countries, we had to compare our findings to other studies that involved adult patients as well.

In conclusion, this study highlights the trends of child and adolescent psychiatric disorders in the last 20 years in a medium developmental region of mainland China, indicating a steady demand for hospitalization of child and adolescent patients with psychiatric disorders, especially those with affective psychiatric disorders. The decline in hospitalization rates for patients with SCZ may indicate the effectiveness of early intervention and introduction of community-based treatment strategies. The increase in the proportion of females admitted for psychiatric disorder inpatients may require further research for targeted early interventions.

## Data availability statement

The raw data supporting the conclusions of this article will be made available by the authors, without undue reservation.

## Ethics statement

The studies involving human participants were reviewed and approved by Ethics Committee of the Fourth People’s Hospital of Hefei, Anhui, China. Written informed consent for participation was not required for this study in accordance with the national legislation and the institutional requirements.

## Author contributions

HoZ, XJ, RY, and SW contributed to the data collection. HoZ contributed to the conceptualization and draft writing. HuZ provided the guidance for the writing of this manuscript. All authors approved the final version for publication.
